# QuartetS-DB: a large-scale orthology database for prokaryotes and eukaryotes inferred by evolutionary evidence

**DOI:** 10.1186/1471-2105-13-143

**Published:** 2012-06-22

**Authors:** Chenggang Yu, Valmik Desai, Li Cheng, Jaques Reifman

**Affiliations:** 1 United States Department of Defense Biotechnology High Performance Computing Software Applications Institute, Telemedicine and Advanced Technology Research Center, U.S. Army Medical Research and Materiel Command, Fort Detrick, MD, 21702, USA

**Keywords:** Orthologs, Orthology detection, Orthology database

## Abstract

**Background:**

The concept of orthology is key to decoding evolutionary relationships among genes across different species using comparative genomics. QuartetS is a recently reported algorithm for large-scale orthology detection. Based on the well-established evolutionary principle that gene duplication events discriminate paralogous from orthologous genes, QuartetS has been shown to improve orthology detection accuracy while maintaining computational efficiency.

**Description:**

QuartetS-DB is a new orthology database constructed using the QuartetS algorithm. The database provides orthology predictions among 1621 complete genomes (1365 bacterial, 92 archaeal, and 164 eukaryotic), covering more than seven million proteins and four million pairwise orthologs. It is a major source of orthologous groups, containing more than 300,000 groups of orthologous proteins and 236,000 corresponding gene trees. The database also provides over 500,000 groups of inparalogs. In addition to its size, a distinguishing feature of QuartetS-DB is the ability to allow users to select a cutoff value that modulates the balance between prediction accuracy and coverage of the retrieved pairwise orthologs. The database is accessible at https://applications.bioanalysis.org/quartetsdb.

**Conclusions:**

QuartetS-DB is one of the largest orthology resources available to date. Because its orthology predictions are underpinned by evolutionary evidence obtained from sequenced genomes, we expect its accuracy to continue to increase in future releases as the genomes of additional species are sequenced.

## Background

The ability to sequence complete genomes is transforming life-science research, especially as it becomes faster, less expensive, and more widely available. Access to this vast amount of genomic data allows us to perform large-scale comparative genomic analyses, so that experimental knowledge can be transferred from well-studied organisms to newly sequenced genomes. Critical to this transfer of knowledge from one organism to another is the concept of homology. Homologous genes evolve from a common ancestral gene, and can be characterized as orthologs or paralogs [1,2]. Orthologous genes evolve through speciation events and, as such, are expected to have retained their molecular functions. Although the functional similarity of orthologs is still being debated [3,4], orthology has been used to infer gene function [5], identify conserved regulatory and functional regions across multiple species [6], predict novel signaling pathways [7], identify common, broad-spectrum protein targets across pathogenic organisms [8], and select pathogen-specific drug targets without human orthologs to minimize potential detrimental effects in patients [9]. Conversely, paralogous genes evolve through duplication events, which offer an opportunity for genes to escape selection pressures and undergo mutations leading to new functional roles [10,11]. Even though, in general, paralogs are not expected to share similar molecular functions, a certain type (termed inparalogs), which evolved from recent duplications subsequent to a given speciation event [1], are also likely to share similar functions. Some orthology databases, such as InParanoid [12], provide within-species inparalog information.

With the recent growth of databases containing complete genome sequences, there has been a parallel growth in the development of new orthology databases, derived from algorithms employing various evolutionary assumptions and approximations. In the past five years, we have observed a steady growth in the number of accessible orthology databases, including many successive updates, where each release covers a larger number of complete genome sequences. For example, OrthoDB’s coverage of complete genomes increased from 57 in its original release to 191 in its last update [13,14], PhylomeDB increased from 443 to 717 [15,16], eggNOG increased from 373 to 630 [17,18], and OMA’s coverage increased nearly 7-fold from 150 to 1000 genomes between 2005 and 2010 [19]. (By the time of this writing, PhylomeDB, eggNOG, and OMA had increased their coverage to 1415, 1133, and 1109 genomes, respectively).

To address the accelerated growth in the number of available genome sequences, orthology detection methods must become more computationally efficient, even when extensive high-performance computational resources are available. One possibility, suggested by Altenhoff et al. [19] is to develop algorithms that bypass portions of the computationally intensive, all-against-all comparative procedure widely used by methods based on bi-directional best hits (BBH). While minimizing such costly pairwise comparisons would be computationally advantageous, care must be taken not to compromise detection accuracy. Hence, the challenge is to develop new methods that can handle large-scale applications (e.g., thousands of genomes) while balancing often diametrically opposed objectives: detection accuracy and computational efficiency.

Recently, we developed a novel orthology detection method, termed QuartetS, that attempts to balance detection accuracy and computational efficiency [20]. QuartetS is a sequence similarity-based orthology prediction method grounded on the well-established evolutionary concept that gene duplication events distinguish orthologous from paralogous relationships [1]. It provides accurate predictions by evaluating whether putative orthologous pairs identified by BLAST analysis as BBH across two genomes have originated from a duplication event. This is typically achieved by analyzing phylogenetic gene trees formed by the two genes of interest and two genes from a third genome, for all available genomes. However, because the construction of phylogenetic trees is computationally intensive, to achieve computational efficiency QuartetS approximates this process via an analytic expression that considers pairwise sequence similarities identified by BLAST. Since evolutionary evidence is extracted from all genomes, we expect the accuracy of QuartetS to continue to increase as additional organisms are sequenced.

In a systematic, large-scale comparison of 624 bacterial genomes using both function- and phylogeny-based metrics [20], we showed that QuartetS slightly, but consistently, outperformed the highly specific Orthologous Matrix (OMA) method [21]. This is notable because in a recent comparative study [21], OMA outperformed a large number of orthology detection methods, and its database is one of the largest of its kind, including orthology predictions from 1000 complete genomes [19].

Here, we present the QuartetS database (QuartetS-DB), which includes orthology predictions among 1621 complete genomes (1365 bacterial, 92 archaeal, and 164 eukaryotic) distributed across 44 phyla (Table 1), covering more than seven million proteins, four million pairwise orthologs, 300,000 orthologous groups, and 500,000 groups of inparalogs. In addition to its size, (arguably the largest database of its kind to date), QuartetS-DB also provides special features for browsing, querying, and downloading orthology information that together may not be readily available elsewhere. These include: *1*) a user-specified cutoff parameter to tailor its application by balancing prediction accuracy and coverage (the user can choose to obtain fewer, more accurate ortholog predictions or more, less accurate ortholog predictions); *2*) the ability to retrieve a list of *all* orthologs across *multiple*, user-specified genomes (a convenient feature for comparative studies); and *3*) the ability to browse more than 236,000 gene trees of the corresponding orthologous groups, including 222 large trees covering over 900 taxa, a desirable feature in evolutionary studies of protein families across species.

**Table 1:** Phylum distribution of the 1621 species in QuartetS-DB

Phylum (Superkingdom)*	**Number of species**
Proteobacteria (B)	669
Firmicutes (B)	303
Actinobacteria (B)	137
Euryarchaeota (A)	60
Bacteroidetes (B)	57
Ascomycota (E)	51
Cyanobacteria (B)	44
Crenarchaeota (A)	28
Tenericutes (B)	27
Chordata (E)	22
Spirochaetes (B)	21
Arthropoda (E)	21
Fusobacteria (B)	15
Chlamydiae (B)	15
Chloroflexi (B)	14
Apicomplexa (E)	13
Thermotogae (B)	11
Chlorobi (B)	11
Streptophyta (E)	10
**Other**	92
**Total**	1621

* Superkingdom: (B) Bacteria, (A) Archaea, (E) Eukarya.

## Construction and content

### The QuartetS method

QuartetS distinguishes paralogs from orthologs using evolutionary evidence of gene duplication events [20]. This evidence is obtained by identifying the location of a putative duplication event in a quartet gene tree formed by the two target genes x and y, and two other genes z1 and z2 in a third species, for all available genomes. If the duplication event is located in the tree segment between genes x and y that overlaps with the path between genes z1 and z2, then x and y are deemed to be paralogs. However, if a search across all species in the database fails to identify such evidence, they are assumed to be orthologs. Rather than inferring the location of a putative duplication event by constructing “precise” gene trees, we bypass this computationally intensive process by deriving an analytic expression that provides an estimate of this location based on pairwise sequence similarities *S*_i,j_, between genes i and j, computed using BLAST bit-scores. The parameter

(1) $$\alpha =\frac{1}{2}\;\left[min\left(S_{x,z1},S_{y,z2}\right)–\frac{1}{4}\;\left(S_{x,z2}+S_{y,z1}+S_{x,y}+S_{z1,z2}\right)\right]$$

provides the likelihood of a duplication event inferred by z1 and z2 along the evolution of the two target genes x and y. Thus, we infer that x and y are orthologs when α is smaller than a specified cutoff value Ω, with a smaller Ω leading to the identification of fewer, more accurate orthologs.

In summary, QuartetS predicts pairwise orthologs by:

1. Identifying putative orthologous pairs x and y flagged by BLAST analysis as BBH across two species;

2. Computing α for a set of gene pairs z1 and z2 in a third species that form BBH with x and y, and computing all possible α for each and every species in a genome database; and

3. Predicting x and y as orthologs if α ≤ Ω, for all α.

This strategy has been shown to produce an acceptable tradeoff between detection accuracy and computational efficiency. The computational cost to fetch the sequence similarity values *S*_i,j_ adds less than 0.5% to the cost of obtaining BBH pairs with BLAST, while the prediction accuracy is similar to that obtained with precisely constructed gene trees [20].

### Acquisition of protein sequence data

We collected the protein sequence data of complete genomes from the NCBI RefSeq database release 43 [22]. This included nearly five million proteins in 1457 prokaryotic species (1365 bacterial and 92 archaeal) and over two million proteins in 164 eukaryotic species. In QuartetS-DB we identified proteins using NCBI GenInfo Identifier (GI) numbers. However, because users may wish to query the database using naming conventions linked to records in other resources, we also downloaded additional protein identifiers, including the NCBI Gene ID, RefSeq accession, Locus tag, and UniProt accession, from the RefSeq database (ftp://ftp.ncbi.nih.gov/refseq/release/release-catalog/) and mapped them to the corresponding protein GI numbers. We obtained protein annotations, such as gene symbol, functional description, and Gene Ontology (GO) terms (GO description and GO accession numbers), from the same site. We named all 1621 species in the database according to RefSeq records and uniquely identified them by their NCBI taxonomy identifier. It should be noted that the RefSeq database contains multiple protein isoforms translated by alternative splicing of the same gene. In QuartetS-DB, we kept all of these isoforms (whose functions might be slightly different) and treated them as separate proteins. This allowed us to identify “orthologous” (functionally similar) protein isoforms in different species.

### Pairwise orthologs

The core product of QuartetS-DB is the collection of pairwise orthologs predicted between each pair of genomes from the 1621 species used in developing the database. To obtain these pairwise orthologs, we first performed an all-against-all BLAST analysis for all seven million proteins from these 1621 genomes to generate BBH pairs and predict putative orthologs. Putative pairwise orthologs are BBH pairs that satisfied two conditions: *1*) the alignment region must cover at least 50% of the length of each sequence, and *2*) the bit-score of the pair alignment had to exceed a cutoff value of 50, which is equivalent to a 10^-5^ E-value cutoff in our database. Currently, QuartetS-DB contains 4.42 million putative pairwise orthologs. We then used the QuartetS method to compute the parameter α for each putative pairwise ortholog and stored the largest value in the database. This allows users to modulate the accuracy of the retrieved pairwise orthologs between two species by specifying a cutoff value, Ω, and only retrieving those orthologs with a corresponding α ≤ Ω. Users can retrieve fewer, but more accurate (i.e., a lower level of false positives) ortholog predictions by specifying a small Ω, or they can retrieve more, but less accurate predictions by specifying a large Ω.

### Orthologous groups

Comparative studies of multiple species require the identification of orthologs across more than two species, which can be inferred from pairwise orthologs by constructing orthologous groups. We constructed more than 300,000 orthologous groups by post-processing the pairwise ortholog predictions (using Ω = 20) and clustering these using the Markov Cluster (MCL) program (version 08–213, downloaded from http://micans.org/mcl). MCL is an unsupervised clustering algorithm [23], previously used by OrthoMCL [24], which clusters pairwise orthologs into orthologous groups, so that each group contains proteins (considered to be orthologs among themselves) from multiple (≥ 3) distinct species.

### Functional annotation for orthologous groups

We employed a simple consensus rule based on the annotations of the individual proteins forming each group to functionally annotate the orthologous groups. When an annotation taxonomy was assigned to more than 80% of the proteins in an orthologous group, that taxonomy, which includes gene symbol, functional description, GO description, and GO accession number, was used to characterize the function of the group. Otherwise, the orthologous group was not annotated with a particular taxonomy. Many orthologous groups were not annotated either because their proteins lacked an annotation in the NCBI RefSeq database or due to inconsistent protein annotations that did not satisfy the consensus rule.

### Gene tree generation for each orthologous group

We constructed gene trees for each orthologous group that contained proteins belonging to four or more species (more than 236,000 gene trees in total). First, we performed multiple sequence alignments of all proteins in a group using muscle (version 3.8.31, accessible at http://www.drive5.com/muscle/) with the default settings. We then used the results to construct gene trees with the RAxML program (version 7.0.4, accessible at http://www.exelixis-lab.org/) using the JTT model. The gene trees are saved in Newick format, so that they can be viewed and exported using the PhyloWidget plug-in [25], and can be downloaded as images or as Newick format text files.

### Inparalog groups

QuartetS-DB also provides inparalog relationships within each one of the 1621 species in the database. These inparalogs are defined as paralogs whose duplication events occurred after the most recent speciation event that separated each species from the other species in the database. Briefly, we constructed a group of inparalogs for a species by evaluating the results of an all-against-all BLAST analysis and identifying a set of proteins in the species such that the alignment and bit-score of any two proteins in the set satisfied the two conditions described in the “Pairwise orthologs” Section and the sequence similarity between any two proteins in the set was higher than the sequence similarity between any one protein in the set and any protein in any one of the other species in QuartetS-DB. We constructed a total of 515,055 groups of inparalogs for the 1621 species. In practice, this information extends the one-to-one pairwise orthology relationships to many-to-many co-orthology relationships because inparalogs of each protein in an orthologous pair are considered to be co-orthologs of the other protein (and its inparalogs) in the pair.

### Database statistics

Table 2 summarizes the content of QuartetS-DB. The database provides over 4.4 million putative pairwise orthologs, including more than four million accurate pairwise orthologs among 1621 species and over seven million proteins. The database also provides more than 300,000 partially annotated orthologous groups, each consisting of orthologous proteins from at least three species, and more than 500,000 groups of inparalogs. For orthologous groups representing four or more species, QuartetS-DB also provides over 236,000 gene trees, including more than 500 gene trees with at least 500 proteins each.

**Table 2:** Summary of the content of QuartetS-DB

**Number of species**	**1621**
Bacteria	1365
Archaea	92
Eukaryotes	164
Fungi	64
Protozoa	33
Invertebrate	30
Mammal	15
Plant	15
Non-mammal vertebrate	7
Number of proteins	7322227
Number of putative pairwise orthologs^1^	4421262
Number of pairwise orthologs (Cutoff Ω = 20)	4022809
Number of orthologous groups (OG)	318618
Number of OGs with gene symbols^2^	78004
Number of OGs with functional descriptions^2^	124074
Number of OGs with GO annotations^2^	54962
Number of gene trees (for OG with size ≥ 4)	236962
Median size of OGs	6
Number of inparalog groups	515055

^1^ Obtained by selecting a large value (10,000) for the QuartetS cutoff Ω, which is equivalent to claiming all BBH pairs as orthologs.

^2^ Based on consensus annotation of the proteins constituting each group.

## Utility and discussion

At its core, QuartetS-DB contains a list of pairwise orthologous proteins, orthologous groups (consisting of orthologous proteins present in more than two species), and inparalog groups. A Web-based interface provides the ability to query the database and to access and download results, which include brief functional descriptions of individual proteins and orthologous and inparalog groups. Additional detailed information for individual proteins is also available via links to external resources, such as NCBI and UniProt. The QuartetS-DB database primarily supports two types of studies: multiple genomes (where the user is interested in identifying a list of orthologs across two or more specific genomes) and individual proteins (where the user is interested in identifying a list of species that contains orthologs of a specific protein).

### Study of multiple genomes

Users may want to identify all orthologs among multiple species to conduct genome-wide comparative studies. For example, when studying human gene functions through mouse models, it is often necessary to obtain human-mouse orthologs so that knowledge about the functions of the mouse genes can be transferred to the corresponding human orthologs. As another example, the identification of orthologs among multiple bacterial genomes is usually the first step in gene association studies involving different phenotypes, such as bacterial pathogenicity and virulence.

**Figure 1:**
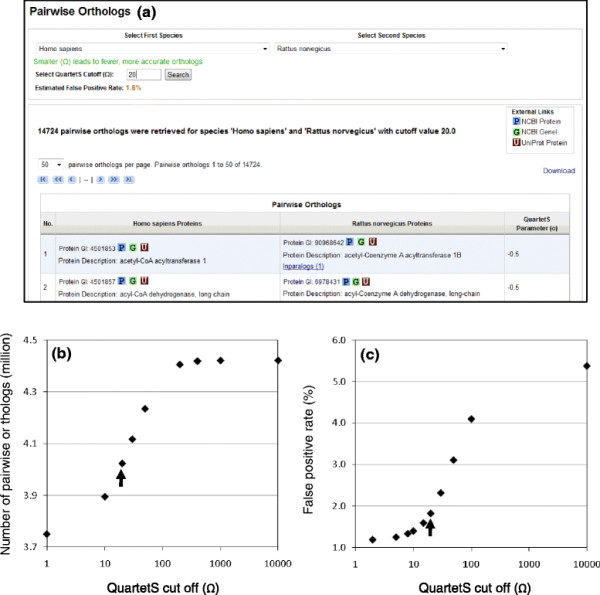
**QuartetS-DB Web Interface and performance curves for “Pairwise Orthologs”.** (**a**) Web interface to search, view, and download pairwise orthologs for any two of 1621 species. By adjusting the QuartetS cutoff value Ω, users can balance the accuracy and coverage of the retrieved pairwise orthologs to support particular applications. (**b**, **c**) Performance curves obtained using varying cutoff values. The false positive rate was estimated using KEGG annotations (KO numbers) for ~900 species. The arrow in each plot indicates the result for the default cutoff value (Ω = 20). Detailed step-by-step instructions for this application can be found within the QuartetS-DB Web site under “User’s Guide”.

The QuartetS-DB Web interface allows users to view and download a list of orthologs between two species via the “Pairwise Orthologs” page of the interface (Figure 1a). Users can select a QuartetS cutoff Ω, to adjust the accuracy of the retrieved pairwise orthologs; selecting a lower cutoff to obtain fewer, more accurate ortholog predictions or selecting a higher cutoff to obtain more, but less accurate predictions. This special function allows the user to tailor the quality of the retrieved results as required. Figures 1b and 1c show the dependency of the number of predicted pairwise orthologs and the associated false positive rate of such predictions, respectively, as a function of the QuartetS cutoff values Ω. The false positive rate was estimated using KEGG annotations (KO numbers) for ~900 species [20]. In the “Pairwise Orthologs” page of the Web interface, when an orthologous protein has inparalogs, a link to these inparalogs is provided, allowing them to be downloaded together with the pairwise orthologs. The inparalogs can also be viewed and downloaded separately via the “Inparalog Groups” page.

**Figure 2:**
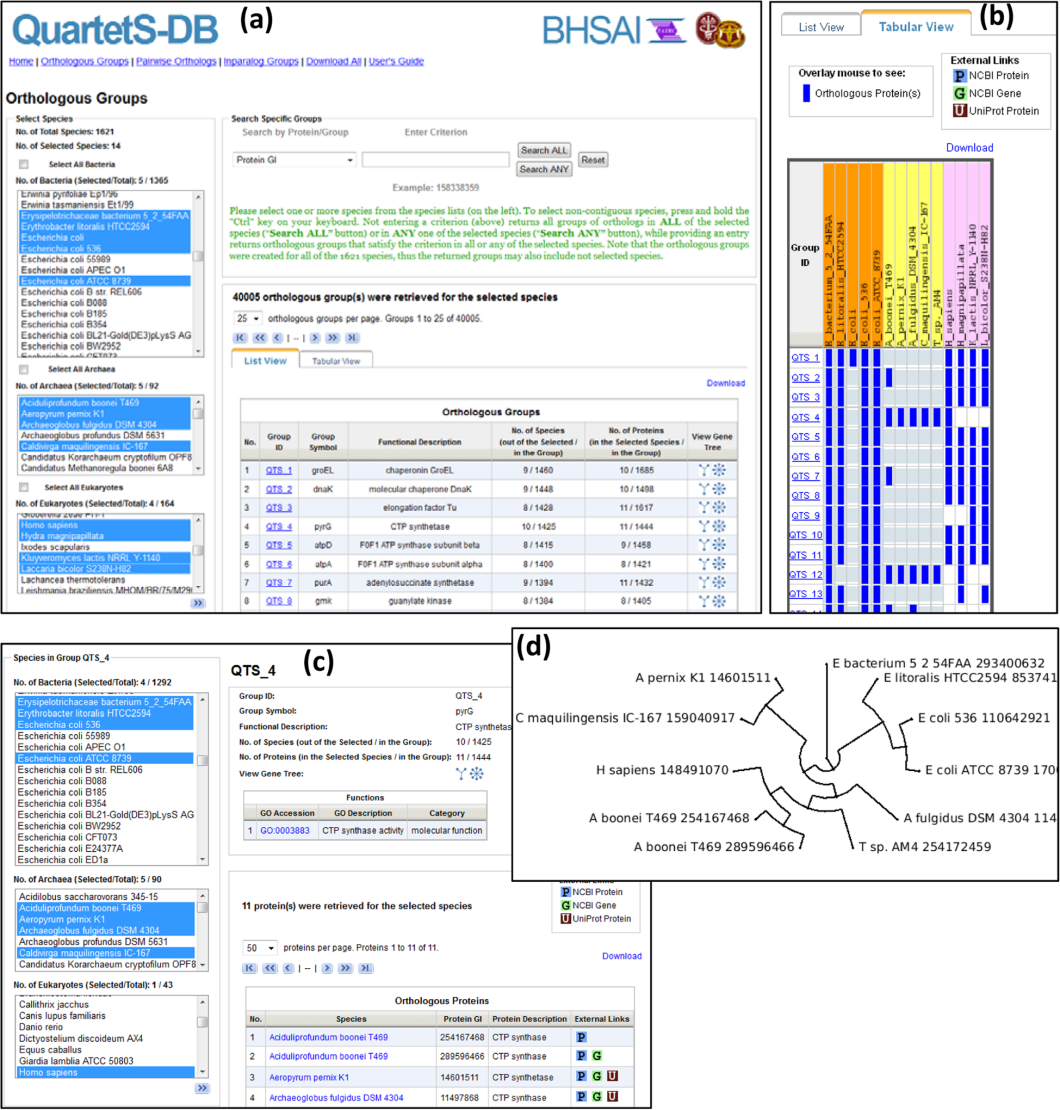
**QuartetS-DB Web interface for “Orthologous Groups”.** (**a**) The interface allows users to query and obtain a list (through “List View”) of orthologous groups. (**b**) The “Tabular View” of multiple orthologous groups indicates whether a group contains an orthologous protein for a given species. (**c**) Detailed information about an orthologous group (e.g., QTS_4), including the orthologous proteins that are contained in the group. (**d**) Display of a pre-computed gene tree for an orthologous group that contains four or more species. Detailed step-by-step instructions for this application can be found within the QuartetS-DB Web site under “User’s Guide”.

**Figure 3:**
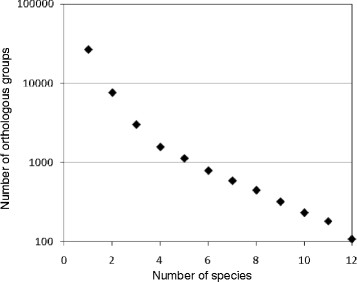
**The number of orthologous groups that contain orthologs in one or more of 12 selected bacterial pathogens ( *Mycobacterium tuberculosis, Bacillus anthracis* Ames *, Listeria monocytogenes, Brucella melitensis* , *Burkholderia mallei* , *Burkholderia pseudomallei* , *Francisella tularensis* , *Coxiella burnetii* , *Legionella pneumophila* , *Salmonella typhimurium* , *Shigella flexneri* , and *Yersinia pestis* ).** A data point for *n* species indicates the number of orthologous groups that contain orthologs in at least *n* of the 12 pathogens.

Orthologs among more than two species can be queried, viewed, and downloaded as orthologous groups from the “Orthologous Groups” page of the QuartetS-DB Web interface (Figure 2), both in “List View” and “Tabular View.” The “List View” (Figure 2a) provides a list of orthologous groups with basic group information, such as the group’s size and functional description. The “Tabular View” (Figure 2b) shows an orthologous group table with each row representing a group and each column representing a species. The entries in the table indicate the presence (blue blocks) of orthologous proteins in the corresponding species (to maintain a concise interface, the “Tabular View” displays a maximum of 100 species). The “Tabular View” is especially useful in comparative genomics studies where the objective is to identify orthologs conserved across multiple species of interest. For example, Figure 3 shows an analysis derived from this information, where we plot the number of orthologous groups that contain orthologs in at least *n* (with *n* = 1, 2, …., 12) of 12 selected bacterial pathogens. The observation that ~100 groups contain orthologs conserved in all 12 pathogens is especially important in studies aiming to identify broad-spectrum antibacterial drug targets. In the “Orthologous Groups” page, the Web interface provides users the option to obtain orthologous groups containing orthologs in any one of the selected species (“Search ANY”) or groups containing orthologs conserved in each one of the selected species (“Search ALL”).

### Study of individual proteins

Users can study individual proteins by using the query function in the “Orthologous Groups” page of the interface. For example, if users would like to infer the function of a specific protein, they can query the protein identifier, such as the protein GI, RefSeq accession, Gene ID, Locus tag, or UniProt accession, to obtain an orthologous group that contains this protein and its orthologs. The annotated orthologous proteins in the group, as well as the group’s functional description (if available), can help to infer the function of the query protein (Figure 2c). The group’s pre-computed gene tree (Figure 2d) may support studies of the evolution of the query protein and its orthologs. All gene trees are displayed using the PhyloWidget plug-in, and can be downloaded as images or as Newick format text files.

In addition to the ability to query a specific protein, users can directly query orthologous groups by their annotations, such as group symbol, functional description, and GO terms. This allows users to find proteins with particular functions and identify species that contain these proteins. For example, if users select two species, *Homo sapiens* and *Mus musculus*, and query groups that contain “telomerase” in the description, they will obtain group QTS_86077 that is described as “telomerase reverse transcriptase” and contains orthologs in the two species. But if they query “dnaC,” no orthologous group is returned, because dnaC is a bacterial protein that has no orthologs in either one of the two species. This feature of QuartetS-DB may be helpful in the selection of pathogen-specific drug targets that do not have human orthologs, so that potential detrimental effects in patients can be minimized.

## Conclusions

By providing access to orthology predictions among 1621 complete genomes based on a recently developed, accurate, and computationally efficient method (QuartetS), QuartetS-DB constitutes one of the largest and reliable resources of its kind. Because its orthology predictions are underpinned by evolutionary evidence obtained from sequenced genomes, we expect its accuracy to continue to increase in future releases as the complete genomes from additional species are sequenced.

QuartetS-DB was primarily designed to support studies involving both the identification of orthologs between two specific species and among genomes of multiple species. For the former, it provides users with the ability to retrieve more than four million accurate pairwise orthologs and to select a cutoff value that modulates the balance between prediction accuracy and coverage of the retrieved pairwise orthologs, satisfying users with distinct needs. For the latter, it provides users with the ability to retrieve a list of *all* orthologs across *multiple*, user-specified genomes and to browse more than 236,000 precomputed gene trees of orthologous groups, both convenient features for comparative studies involving multiple genomes.

## Availability and requirements

The database is freely accessible at https://applications.bioanalysis.org/quartetsdb. The Web interface operates with various browsers, including Safari, Internet Explorer 7 or later, Firefox 8 or later, and Google Chrome. We used the PhyloWidget Java Applet to display gene trees, which requires Sun Java 1.5 or greater. Detailed system requirements for this Java Applet can be found at the PhyloWidget Website (http://www.phylowidget.org). There are no other system requirements. We plan to update the database annually.

## Competing interest

The authors declare that they have no competing interests. The opinions and assertions contained herein are the private views of the authors and are not to be construed as official or as reflecting the views of the U.S. Army or the U.S. Department of Defense. This paper has been approved for public release with unlimited distribution.

## Authors’ contributions

CY developed the QuartetS algorithm. VD and LC developed the QuartetS-DB database and VD developed the Web interface for the database. CY and JR conceived the work and wrote the manuscript. All authors read and approved the final manuscript.
